# Factors associated with clinical breast examination and cervical cancer screening uptake in Ghanaian women, Evidence from the 2022 Ghana Demographic and Health Survey

**DOI:** 10.4314/ahs.v25i3.17

**Published:** 2025-09

**Authors:** Maxwell Akonde, Joseph Anyinka, Georgina Abojoka Abah, Rajat Das Gupta, Bernard Frempong, Cynthia Cupit Swenson

**Affiliations:** 1 University of South Carolina Arnold School of Public Health, Department of Epidemiology and Biostatistics Columbia, SC, US 29208-0001; 2 University of Ghana, Department of Geography and Resource Development, Legon, Greater Accra, Ghana; 3 Bingham University College of Health Sciences, Abuja-Keffi Rd, Karu, Nasarawa, Nigeria; 4 University of North Dakota, School of Medicine and Health Sciences, Department of Biomedical Sciences, Grand Folks, ND, 58202, USA; 5 Medical University of South Carolina Division of Global and Community Health, 176 Croghan Spur, Suite 104 Charleston, SC, US 29425-2503

**Keywords:** Screening, breast, cervical, cancer, Ghana, wealth index

## Abstract

**Background:**

Breast and cervical cancer screening uptake remains limited in Ghana despite the public health importance of cancer screening. This study examines factors that influence this behavior.

**Methods:**

Secondary analyses of the 2022 Ghana Demographic and Health Survey (GDHS) were conducted. A total of 9,489 women between the ages of 25 to 49 years were evaluated for clinical breast examination (CBE) and cervical cancer screening (CCS) uptakes. Univariable and multivariable logistic regressions were fitted to examine factors that influence women utilization of CBE and CCS.

**Results:**

Women in the richer and richest quintiles of wealth index were 40% (AOR=1.40; 95%CI: 1.00 – 1.97) and 118% (AOR=2.18; 95%CI: 1.51 – 3.16) significantly more likely to attain CBE compared to women in the poorest quintile. Women with secondary and higher education were 2.02 times (AOR=2.02; 95%CI: 1.59 – 2.56) and 4.94 times (AOR=4.94; 95%CI: 3.61 – 6.76) as likely to attain CBE compared to women with no education. Women with health insurance were 85% (AOR=1.85; 95%CI: 1.37 – 2.52) more likely to attain CBE compared to women without health insurance. Similar results were found on the associations between wealth index, educational level and health insurance status and CCS uptake.

**Conclusion:**

CBE and CCS uptakes were significantly higher in women with higher wealth index, higher education, and health insurance highlighting potential disparities in access to and utilization of preventive services in Ghanaian women. Tailoring policies to address the poverty burden will likely lead to an increase in breast and cervical cancer screening uptakes and potentially better health outcomes.

## Introduction

Breast and cervical cancers are the first and fourth most common cancers in women globally^1^,^2^. Both cancers are leading causes of cancer deaths in women worldwide with disproportionately higher mortality and morbidity burden occurring in low-and-middle income countries (LMICs) 1–5. In Ghana, breast and cervical cancers are significant public health concerns ranking among higher causes of cancer related deaths in women^6^,^7^. Although effective screening programs for early detection that lead to reduce mortality rates in these cancers exist, uptake of these programs remains low in Ghana^7^,^8^.

With nearly 30% of breast cancers occurring in women under the age of 35 years in Ghana^7^, breast screening is critical to improving survival. Whilst mammography and ultrasound of the breast may not be available in all health facilities^9^, clinical breast examination (CBE) can be achieved in most health facilities across the country prompting the need to study why clinical breast examination uptake remains low. Similarly, the low uptake of pap smear and/or testing for human papilloma virus^10^,^11^ raises concerns of the increasing burden of cervical cancer in LMIC including Ghana and suggests the need to better classify factors that impact the cervical cancer screening (CCS) uptake.

Understanding factors that influence CBE and CCS uptake is important for designing effective interventions tailored towards improving screening uptake and reducing the burden of these cancers. Some studies have reported the association between sociodemographic factors and cancer screening uptakes. For example, older age was found to be associated with higher uptake of breast and cervical cancer screening^12^-^14^. Women with higher education and socioeconomic status have also been reported to have increased likelihood of cancer screening uptake^12^,^14^. Lastly, women who identified as married or living with partners also reported having a positive attitude for cancer screening uptake compared to women who were not married or divorced^15^.

Although some evidence on factors that influence breast or cervical cancer screening rates has been reported in Ghana, most of the studies^7^,^16^-^20^ are limited to specific regions, cities or were hospital-based designs limiting the generalizability of these studies to the Ghanaian population. Additionally, these studies are limited by the small sample size. The Ghana Demographic and Health Survey (GDHS) serves as a valuable resource that has the potential to address these limitations. Our study, therefore, evaluates the associations between sociodemographic, lifestyle and clinical related factors and clinical breast examination uptake using the nationally representative data of the GDHS. The study also evaluates the associations between sociodemographic, lifestyle and clinical related factors and cervical cancer screening in the Ghanaian population.

## Methods

This study utilized data from the 2022 Ghana Demographic Health Survey (GDHS). This is a nationally representative survey that was conducted between October 17, 2022, and January 14, 2023. Details of the survey were previously published^21^. To provide a summary, the 2022 GDHS was implemented by the Ghana Statistical Services that collected data from approximately 18,540 households in 618 selected clusters across 16 regions. The sampling framework that used the 2021 Population and Housing Census was a stratified two-stage cluster sampling designed to yield results at the national level for urban and rural areas, and for each region for most of the GDHS indicators. A probability proportional to size (PPS) for urban and rural areas in each region was applied in the first stage to select the 618 target clusters followed by the targeted cluster number selected with equal probability, systematic random sampling of the clusters selected in the first phase. A household listing and map updating operation was conducted on the selected clusters to develop a list of all the households in the clusters for the second stage. The list generated served as the framework for selecting participating households. A fixed number of 30 households were randomly selected from each cluster. Four questionnaires from the Demographic and Health Survey Program's Model Questionnaires were adapted to reflect the health and population issues of Ghana. These included household, woman, man, and biomarker questionnaires. The present study was limited to the data collected with the women's questionnaire as breast and cervical cancers are predominantly cancers affecting women. This questionnaire was administered to 15,014 women between the ages 15 – 49 years. Since the recommended start age for clinical breast examination and cervical cancer screening is 25 years^22^,^23^, a total of 9,489 women who were aged between 25 – 49 years were included in this study.

### Study variables

#### Dependent variables

The dependent variables were clinical breast examination (CBE) and cervical cancer screening (CCS). CBE was assessed by asking the question “Has a doctor or other healthcare provider examined your breasts to check for breast cancer?” with responses “No” and “Yes”. CCS was assessed by asking the question “Has a doctor or other healthcare worker ever tested you for cervical cancer?” with the responses “No” and “Yes”.

#### Independent variables

The independent variables considered in this study included: wealth index, age, distance to nearest health facility, means of transport, education level, marital status, health insurance, region of residence, rural-urban, religion, work, contraceptive use, use of bed net, age of coitarche and number of sex partners. These variables were selected by reviewing literature^12^-^20^ and their availability in the DHS. Details of the description and categorization of the variables are presented in supplementary Table 1.

**Supplementary Table 1A:** Description of selected variables in the 2022 Ghana Demographic and Health Survey Use in this study

Variable	Description	Categories
Dependent		
Clinical breast examination	Has a doctor or other healthcare provider examined your breasts to check for breast cancer?	No; Yes
Cervical cancer screening	Has a doctor or other healthcare worker ever tested you for cervical cancer?	No; Yes
*Independent*		
Wealth index	Assessed as a composite measure of a household's living standard. It was calculated using data on household ownership of selected assets such as televisions and bicycles as well as materials used for housing construction and access to water and sanitation facilities.	Poorest; Poorer; Middle; Richer; and Richest
Age	Current age in years	25 – 34 years; 35 – 44 years; ≥45 years
Distance to nearest health facility	How long does it take in minutes to go from your home to the nearest healthcare facility, which could be a hospital, a health clinic, a medical doctor, or a health post?	≤10 minutes; 11 – 20 minutes; 21 – 30 minutes; 31 – 60 minutes; and >60 minutes
Means of transport to health facility	How do you travel to this healthcare facility from your home?	Motorized (car/truck, public bus, motorcycle/scooter, or boat with motor); Non-motorized (animal-drawn cart, bicycle, boat without motor or walking)
Education	Have you ever attended school? and What is the highest level of school you attended: primary, secondary, or higher?	No education; Basic education; Secondary education; and Higher education
Marital status	Are you currently married or living together with a man as if married?; Have you ever been married or lived together with a man as if married?; and What is your marital status now: are you widowed, divorced, or separated?	Never married; Formerly married; and Currently married
Health insurance	Whether participants had any type of health insurance	No; Yes
Region of residence	This was the region where the participant was interviewed	Greater Accra, Ashanti, Western, Central, Eastern, Western North, Ahafo, Bono, Bono East, Volta, Otti, Northern, Savannah, North East, Upper East and Upper West regions and recoded using the multidimensional poverty index (MPI) as Low MPI regions (Greater Accra and Ashanti regions); Medium MPI regions (Western, Central, Eastern, Western North, Ahafo, Bono and Bono East regions); High MPI regions (Volta, Otti, Northern, Savannah, North East, Upper East and Upper West regions) (24).
Rural-urban	Whether they resided in a rural or urban areas	Rural; Urban
Religion	What is your religion?	Christianity; Islam; Others
Work	Have you done any work in the last 12 months?	No; Yes
Contraceptive use	Ever used anything or tried to delay or avoid getting pregnant?	Never; Ever
Use of bed net	Participants were asked whether they had bed net for sleeping	No; Yes
Age of coitarche	Age at which participants had their first sexual intercourse	Never; ≤ 15 years; 16 – 19 years; ≥20 years
Number of sex partners	The number of sex partners a participant had in the last 12 months	Never had sex; One partner; Multiple partners

### Statistical analysis

Weighted analyses were conducted using the Taylor series linearization variance estimation of standard errors method (25). The respondents' characteristics were summarized as frequencies and weighted percentages for categorical variables and as means and standard deviations for continuous variables. The summary statistics were stratified by their clinical breast examination (“No” versus “Yes”) and cervical cancer screening (“No” versus “Yes”) status. Rao-Scott Chi-square statistics were used to examine the differences in the distribution of each characteristic across the CBE and CCS status for the categorical variables whilst an independent two sample t-test was used to assess the differences in distribution for the continuous variables across the CBE and CCS status. The regional distribution of CBE and CCS uptake in percentage was also examined by plotting the proportion of CBE and CCS uptake by respondent's region of residence on the map of Ghana using ARCGIS 10.8.2 geographical information system software (26).

The association between respondents' characteristics and clinical breast examination and between the respondents' characteristics and cervical cancer screening were assessed by fitting separate univariable and multivariable logistic regression models. The regression models accounted for the cluster sampling of the study design. In the multivariable logistic regressions, variables that had a p-value<0.2 in the univariable models and variables that were previously found to be associated with cancer screening uptake were included in the final model. For each independent variable, other covariates were considered confounders and adjusted for. Crude odds ratios and adjusted odds ratios with their 95% confidence interval were computed for each independent variable and the dependent variables. A p-value <0.05 was considered statistically significant unless stated. All the analyses were conducted using SAS version 9.4 unless indicated.

## Results

The women averaged 35.55 years old with most of them (47.66%) being between the ages of 25 – 34 years. The women spent a median time of 27.76 minutes in travel to the nearest health facility. The women mostly used non-motorized transport (58.45%) travelling to the nearest health facility. More than half of the women (50.68%) had secondary school education with nearly three-quarters (72.59%) currently married. Most of the women (57.57%) lived in urban areas and Christianity was the most predominant religion (76.60%). Over 90% reported having health insurance, 73.88% reported using a bed net, and 67.19% reported ever using contraceptives. Additionally, 82.27% reported having one sex partner whilst most of the women (51.97%) had an age of coitarche between 16 -19 years (Table 1).

**Table 1:** Characteristics of the Women in the Ghana Demographic and Health Survey 2022 between 25 – 49 years

		Clinical Breast Examination			Cervical Cancer Screening		
Variable	Total N=9489 %=100	No N=7573 %=77.45	Yes N=1916 %=22.55	P-value	No N=8898 %=93.14	Yes N=591 %=6.86	P-value
Age, years (Means, SD)	35.55(6.96)	35.47(6.89)	35.80(7.20)	0.0558	35.48(6.94)	36.39(7.14)	0.0012
Age in categories, years							
25 – 34	4606(47.66)	3652(48.11)	954(46.10)	0.4263	4323(47.98)	283(43.17)	0.0924
35 – 44	3657(38.73)	2935(38.51)	722(39.48)		3437(38.64)	220(39.98)	
>=45	1226(13.61)	986(13.39)	240(14.42)		1138(13.38)	88(16.85)	
Distance from nearest health facility, minutes (Median, range)	27.76(0 – 600)	28.98(0 – 600)	23.56(0 – 240)	<0.0001	28.25(0 – 600)	21.13(0 – 180)	<0.0001
Distance from nearest health facility (categories), minutes							
<=10	2836(30.92)	2146(29.71)	690(35.10)	**<0.0001**	2604(30.05)	232(42.72)	**<0.0001**
11 – 20	2290(25.26)	1798(24.79)	492(26.88)		2157(25.37)	133(23.70)	
21 – 30	2071(22.49)	1660(22.38)	411(22.86)		1948(22.70)	123(19.56)	
31 – 60	1721(16.49)	1464(17.74)	257(12.22)		1637(16.87)	84(11.29)	
>60	571(4.84)	505(5.39)	66(2.94)		552(4.99)	19(2.73)	
Wealth index quintiles							
Poorest	2262(15.94)	2040(18.56)	222(6.92)	**<0.0001**	2207(16.76)	55(4.67)	**<0.0001**
Poorer	2034(17.14)	1760(19.04)	274(10.65)		1962(17.78)	72(8.48)	
Middle	1829(19.64)	1497(20.74)	332(15.85)		1733(19.97)	96(15.15)	
Richer	1757(22.81)	1333(22.57)	424(23.65)		1616(22.68)	141(24.67)	
Richest	1607(24.47)	943(19.09)	664(42.94)		1380(22.81)	227(47.03)	
Education level							
No education	2882(12.58)	2607(25.55)	275(9.59)	**<0.0001**	2800(22.80)	82(10.40)	**<0.0001**
Primary	1431(14.70)	1233(16.24)	198(9.39)		1373(15.12)	58(8.89)	
Secondary	4120(50.68)	3198(49.81)	922(53.66)		3877(51.15)	243(44.27)	
Higher	1056(12.67)	535(8.40)	521(27.36)		848(10.92)	208(36.44)	
Marital status							
Never married	1002(12.81)	754(12.36)	248(14.35)	0.1547	934(12.90)	68(11.56)	0.7258
Formerly married	1159(14.60)	939(14.98)	220(13.28)		1089(14.54)	70(15.32)	
Currently married	7328(72.59)	5880(72.66)	1448(72.37)		6875(72.55)	453(73.12)	
Health insurance							
No	745(8.82)	671(9.99)	74(4.80)	**<0.0001**	724(9.17)	21(4.00)	**<0.0001**
Yes	8744(91.18)	6902(90.01)	1842(95.20)		8174(90.83)	570(96.00)	
Region							
Low MPI	1383(35.96)	1026(26.54)	357(41.78)	**<0.0001**	1278(35.51)	105(42.08)	0.1550
Medium MPI	3854(38.45)	2989(38.11)	865(39.62)		3627(38.77)	227(34.09)	
High MPI	4252(25.59)	3558(27.62)	694(18.60)		3993(25.72)	259(23.83)	
Rural-urban							
Rural	4795(42.43)	4099(46.46)	696(28.62)	**<0.0001**	4607(43.72)	188(24.93)	**<0.0001**
Urban	4694(57.57)	3474(53.54)	1220(71.38)		4291(56.28)	403(75.07)	
Religion							
Christianity	6541(76.50)	5047(74.37)	1494(83.84)	**<0.0001**	6091(76.15)	450(81.32)	**0.0026**
Islam	2509(19.22)	2114(20.53)	395(14.70)		2375(19.34)	134(17.55)	
Others	439(4.28)	412(5.10)	27(1.46)		432(4.51)	7(1.13)	
Transport							
Non-motorized	5858(58.45)	4789(61.33)	1069(53.01)	**<0.0001**	5527(59.82)	331(54.45)	0.0895
Motorized	3631(40.55)	2784(38.67)	847(46.99)		3371(40.18)	260(45.55)	
Contraceptive use							
Never	3272(32.81)	2713(33.53)	559(30.35)	0.0604	3100(33.06)	172(29.46)	0.1920
Ever	6217(67.19)	4860(66.47)	1357(69.65)		5798(66.94)	419(70.54)	
Work							
No	1323(12.36)	1092(13.00)	231(10.15)	**0.0062**	1256(12.57)	67(9.44)	**0.0315**
Yes	8166(87.64)	6481(87.00)	1685(89.85)		7642(8-7.43)	524(90.56)	
Use of bed net							
No	2210(26._5_2)	1760(26.09)	450(26.22)	0.92833	2061(25.66)	149(32.32)	**0.0098**
Yes	7279(73.88)	5813(73.91)	1466(73.78)		6837(74.34)	442(67.68)	
Age of coitarche, years							
Never had sex	80(1.29)	59(1.27)	21(1.34)	**<0.0001**	75(1.32)	5(0.87)	**<0.0001**
≤15	2160(22.42)	1840(23.89)	320(17.38)		2068(22.75)	92(17.95)	
16 – 19	5055(51.97)	4079(52.78)	976(49.20)		4774(52.39)	281(46.36)	
≥20	2194(23.32)	1595(22.06)	599(32.08)		1981(23.54)	213(34.82)	
Number of sex partners							
Never had sex	1411(16.02)	1124(15.98)	287(16.14)	0.8500	1329(16.04)	82(15.75)	0.9526
1	7917(82.27)	6321(82.36)	1596(81.96)		7419(82.24)	498(82.72)	
2	161(1.71)	128(1.66)	33(1.90)		150(1.73)	11(1.53)	

SD=standard deviation; MPI=Multidimensional Poverty Index; Categorical variables are presented as frequencies with weighted percentages

When the women's characteristics were compared between CBE status, distance to nearest health facility (P-value<0.0001), wealth index (P-value<0.0001), education status (P-value<0.0001), health insurance (P-value<0.0001), region of residence (P-value<0.0001), rural-urban location (P-value<0.0001), religion (P-value<0.0001), means of transportation (P-value<0.0001), work status (p-value=0.0062), and age of coitarche (P-value<0.0001) significantly differed between women who had CBE and women who did not (Table 1). For example, 35.10% women who had CBE travelled 10 or less minutes to the nearest health facility compared to only 29.71% of women who did not have CBE. Also, only 9.59% of the women who had CBE had no education compared to 25.55% of women who did not have CBE. Almost three-quarters of the women who had CBE lived in urban are as compared to only about half of the women who did not have CBE. Fewer women who had CBE (17.38%) were likely to have age of coitarche at 15 or less years compared to women who did not have CBE (23.89%).

In terms of CCS, age (p-value=0.0012), distance to nearest health facility, wealth index (P-value<0.0001), education status (P-value<0.0001), health insurance (P-value<0.0001), rural-urban location (P-value<0.0001), religion (P-value=0.0026), work status (p-value=0.0315), use of bed net (p-value=0.0098) and age of coitarche (P-value<0.0001) significantly differed between women who had CCS and women who did not (Table 1). For example, the women who had CCS were relatively older (36 years) than those who did not (35 years). The number of women who had CCS who were in the richest wealth index group were almost twice (47.03% vs 22.81%) the women who did not have CCS who were in the richest wealth index group.

**Figure 1:**
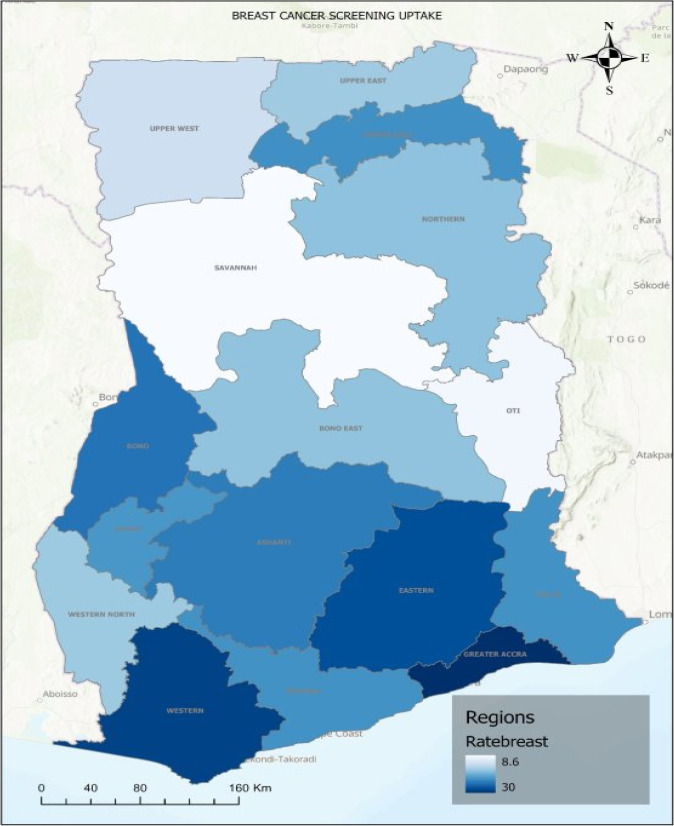
Distribution of clinical breast examination uptake across the 16 regions of Ghana according to the 2022 Ghana Demographic and Health Survey for women between 25 – 49 years; Ratebreast is defined by the percentage of women who undertook the clinical breast examination

When the distribution of CBE and CCS uptakes were examined across respondents' regions, the highest uptake of CBE was observed in the Greater Accra region (29.6 per 100 women) followed by Western (28.1 per 100 women), Eastern (27.1 per 100 women), and Bono (24.1 per 100 women) regions. The lowest CBE uptake was observed in the Savannah region (8.6 per 100 women) followed by Otti region (9.3 per 100 women) (Figure 1).

**Figure 2:**
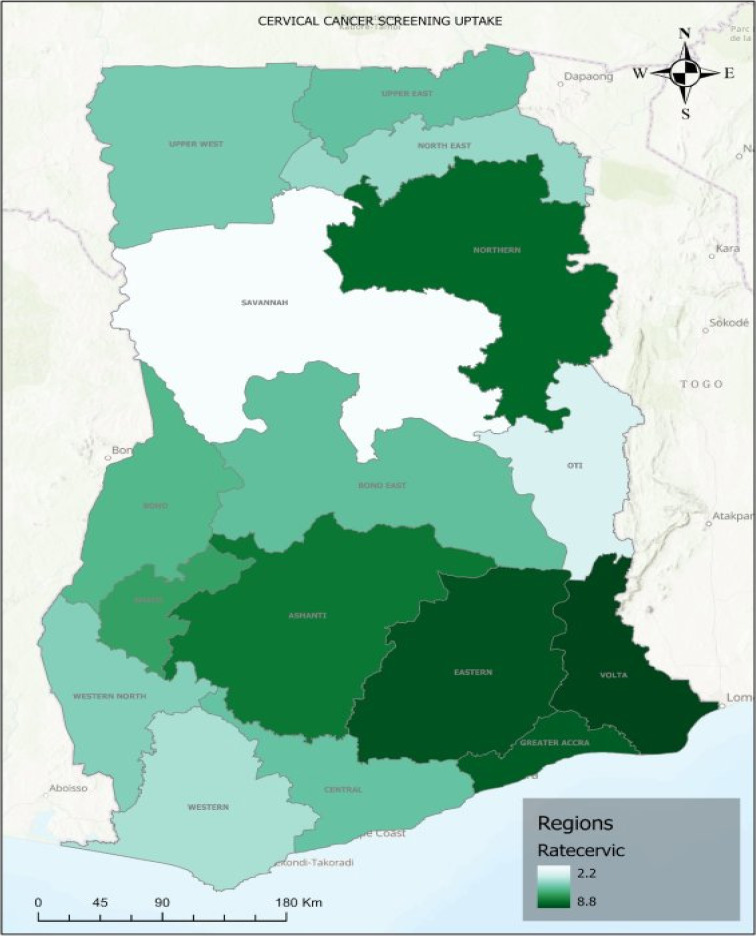
Distribution of cervical cancer screening uptake across the 16 regions of Ghana according to the 2022 Ghana Demographic and Health Survey for women between 25 – 49 years; Ratecervic is defined by the percentage of women who undertook the cervical cancer screening

The highest uptake of CCS was observed in the Volta region (8.8 per 100 women) followed by Eastern (8.5 per 100 women), Greater Accra (8.3 per 100 women) and Northern (8.1 per 100 women) regions. Again, the lowest uptake CCS was observed in the Savannah region (2.2 per 100 women) followed by the Otti region (3.4 per 100 women) (Figure 2).

Table 2 presents the results of the regression analyses. In the adjusted models examining the associations between the women's characteristics and CBE, older women were more likely to get a CBE compared to younger women (35 – 44 years versus 25 -34 years: AOR=1.23; 95%CI: 1.06 – 1.42; 45 or more years versus 25 -34 years: AOR=1.59; 95%CI: 1.26 – 2.00). The women who were in the richer and richest quintiles of wealth index were 40% (AOR=1.40; 95%CI: 1.00 – 1.97) and 118% (AOR=2.18; 95%CI: 1.51 – 3.16) significantly more likely to get CBE compared to women in the poorest quintile. Women who had secondary and higher education were 2.02 times (AOR=2.02; 95%CI: 1.59 – 2.56) and 4.94 times (AOR=4.94; 95%CI: 3.61 – 6.76) as likely to get CBE compared to women who had no education. Women who had health insurance were 85% (AOR=1.85; 95%CI: 1.37 – 2.52) more likely to get CBE compared to women who had no health insurance. Women who had work and women who use bed nets were 26% (AOR=1.26; 95%CI: 1.01 – 1.59) and 23% (AOR=1.23; 95%CI: 1.04 – 1.44) more likely to get CBE compared to women who had no work and women who did not use bed nets respectively. Women who identified with other religions (except Islam) were 50% (AOR=0.50; 95%CI: 0.29 – 0.85) less likely to get CBE compared to women who identified as Christians. All other characteristics were not statistically significant.

When the associations between the women's characteristics and CCS were examined, women who were 45 years or older were 94% (AOR=1.94; 95%CI: 1.37 – 2.73) more likely to get CCS compared to women who were 25 – 34 years. Women who were 11 – 20 minutes away from the nearest health facility were 29% (AOR=0.71; 95%CI: 0.53 – 0.95) less likely to get CCS compared to women who were 10 minutes or less from the nearest health facility. By wealth index, women who were middle, richer, and richest quintiles of wealth index were 2.16 times (AOR=2.16; 95%CI: 1.31 – 3.56), 2.61 times (AOR=2.61; 95%CI: 1.55 – 4.41) and 3.27 times (AOR=3.27; 95%CI: 1.81 – 5.92) as likely to get CCS compared to women who poorest. Also, women who had higher education were 4.58 times (AOR=4.58; 95%CI: 2.73 – 7.68) as likely to get CCS compared to women who had no education. Women who were formerly married and women who were currently married were 1.71 times (AOR=1.71; 95%CI: 1.05 – 2.76) and 1.51 times (AOR=1.51; 95%CI: 1.05 – 2.19) as likely to get CCS compared to women who were never married. Women who reported having health insurance were 97% (AOR=1.97; 95%CI: 1.17 – 3.31) more likely to get CCS compared to women who did not have health insurance. All other characteristics were not statistically significant.

**Table 2:** Predictors associated with clinical breast examination and cervical cancer screening in Ghanaian women between 25 – 49 years

Variable	Clinical Breast Examination		Cervical Cancer Screening	
	COR	AOR	COR	AOR
**Age, years**				
25 – 34	1.00 (Reference)	1.00 (Reference)	1.00 (Reference)	1.00 (Reference)
35 – 44	1.07(0.94 – 1.22)	**1.23(1.06 – 1.42)**	1.15(0.92 – 1.43)	1.26(0.98 – 1.61)
>=45	1.13(0.92 – 1.38)	**1.59(1.26 – 2.00)**	1.4(1.03 – 1.90)	**1.94(1.37 – 2.73)**
**Distance from nearest health facility, minutes**				
<=10	1.00 (Reference)	1.00 (Reference)	1.00 (Reference)	1.00 (Reference)
11 – 20	0.92(0.76 – 1.10)	0.98(0.82 – 1.18)	0.66(0.49 – 0.88)	**0.71(0.53 – 0.95)**
21 – 30	0.87(0.71 – 1.06)	0.98(0.80 – 1.22)	0.61(0.44 – 0.83)	0.73(0.52 – 1.03)
31 – 60	0.58(0.47 – 0.73)	0.87(0.68 – 1.10)	0.47(0.32 – 0.69)	0.74(0.50 – 1.11)
>60	0.46(0.31 – 0.68)	0.90(0.60 – 1.33)	0.38(0.16 – 0.92)	0.85(0.37 – 1.96)
**Wealth index quintiles**				
Poorest	1.00 (Reference)	1.00 (Reference)	1.00 (Reference)	1.00 (Reference)
Poorer	1.50(1.16 – 1.95)	1.11(0.87 – 1.41)	1.71(1.11 – 2.63)	1.45(0.94 – 2.24)
Middle	2.05(1.56 – 2.69)	1.24(0.89 – 1.72)	2.73(1.63 – 4.56)	**2.16(1.31 – 3.56)**
Richer	2.81(2.14 – 3.69)	**1.40(1.00 – 1.97)**	3.91(2.40 – 6.36)	**2.61(1.55 – 4.41)**
Richest	6.04(4.76 – 7.66)	**2.18(1.51 – 3.16)**	7.41(4.77 – 11.49)	**3.27(1.81 – 5.92)**
**Education level**				
No education	1.00 (Reference)	1.00 (Reference)	1.00 (Reference)	1.00 (Reference)
Primary	1.54(1.16 – 2.06)	1.25(0.94 – 1.67)	1.29(0.78 – 2.15)	1.04(0.64 – 1.70)
Secondary	2.87(2.34 – 3.53)	**2.02(1.59 – 2.56)**	1.90(1.32 – 2.73)	1.41(0.96 – 2.07)
Higher	8.68(6.74 – 11.18)	**4.94(3.61 – 6.76)**	7.32(4.80 – 11.15)	**4.58(2.73 – 7.68)**
**Marital status**				
Never married	1.00 (Reference)	1.00 (Reference)	1.00 (Reference)	1.00 (Reference)
Formerly married	0.76(0.57 – 1.03)	0.99(0.70 – 1.38)	1.18(0.78 – 1.77)	**1.71(1.05 – 2.76)**
Currently married	0.86(0.71 – 1.05)	1.19(0.95 – 1.49)	1.13(0.81 – 1.56)	**1.51(1.05 – 2.18)**
**Health insurance**				
No	1.00 (Reference)	1.00 (Reference)	1.00 (Reference)	1.00 (Reference)
Yes	2.20(1.62 – 2.99)	**1.85(1.37 – 2.52)**	2.42(1.44 – 4.08)	**1.97(1.17 – 3.31)**
**Region of residence by MPI**				
Low MPI	1.00 (Reference)	1.00 (Reference)	1.00 (Reference)	1.00 (Reference)
Medium MPI	0.85(0.70 – 1.04)	1.16(0.94 – 1.44)	0.74(0.54 – 1.03)	1.01(0.73 – 1.42)
High MPI	0.55(0.44 – 70)	1.07(0.84 – 1.37)	0.78(0.54 – 1.14)	**1.50(1.03 – 2.19)**
**Rural-urban areas**				
Rural	1.00 (Reference)	1.00 (Reference)	1.00 (Reference)	1.00 (Reference)
Urban	2.16(1.81 – 2.59)	**1.32(1.07 – 1.62)**	2.34(1.76 – 3.11)	1.27(0.93 – 1.74)
**Religion**				
Christianity	1.00 (Reference)	1.00 (Reference)	1.00 (Reference)	1.00 (Reference)
Islam	0.64(0.52 – 0.77)	0.93(0.76 – 1.13)	0.85(0.62 – 1.17)	1.07(0.78 – 1.47)
Others	0.25(0.15 – 0.43)	**0.50(0.29 – 0.85)**	0.24(0.08 – 0.68)	0.42(0.13 – 1.29)
**Means of transportation**				
Non-motorized	1.00 (Reference)	1.00 (Reference)	1.00 (Reference)	1.00 (Reference)
Motorized	1.41(1.19 – 1.66)	1.12(0.96 – 1.30)	1.25(0.97 – 1.60)	0.97(0.76 – 1.25)
**Contraceptive use**				
Never	1.00 (Reference)	1.00 (Reference)	1.00 (Reference)	1.00 (Reference)
Ever	1.16 (0.99 – 1.35)	1.05(0.89 – 1.24)	1.18(0.91 – 1.54)	1.09(0.80 – 1.49)
**Work**				
No	1.00 (Reference)	1.00 (Reference)	1.00 (Reference)	1.00 (Reference)
Yes	1.32(1.07 – 1.63)	**1.26(1.01 – 1.59)**	1.38(1.00 – 1.91)	1.32(0.91 – 1.91)
**Use of bed net**				
No	1.00 (Reference)	1.00 (Reference)	1.00 (Reference)	1.00 (Reference)
Yes	0.99(0.85 – 1.16)	**1.23(1.04 – 1.44)**	0.72(0.58 – 0.91)	0.85(0.67 – 1.08)
**Age of coitarche, years**				
Never had sex	1.00 (Reference)	1.00 (Reference)	1.00 (Reference)	1.00 (Reference)
≤15	0.69(0.38 – 1.27)	1.85(0.86 – 3.98)	1.20(0.41 – 3.52)	2.49(0.73 – 8.52)
16 – 19	0.89(0.48 – 1.62)	1.85(0.86 – 3.97)	1.34(0.49 – 3.70)	2.14(0.65 – 7.02)
≥20	1.38(0.74 – 2.59)	1.95(0.92 – 4.15)	2.24(0.79 – 6.33)	2.25(0.70 – 7.29)
**Number of sex partners**				
Never had sex	1.00 (Reference)	1.00 (Reference)	1.00 (Reference)	1.00 (Reference)
1	0.99(0.82 – 1.19)	0.87(0.70 – 1.09)	1.02(0.74 – 1.42)	0.94(0.61 – 1.47)
2	1.14(0.68 – 1.89)	1.24(0.72 – 2.13)	0.91(0.38 – 2.15)	1.03(0.44 – 2.41)

MPI=Multidimensional Poverty Index; COR=crude odds ratio; AOR=Adjusted odds ratio

## Discussion

This study examined how sociodemographic, lifestyle, and health related characteristics of women are associated with clinical breast examination (CBE) and cervical cancer screening (CCS). Overall, CBE and CCS uptakes were 22.55% and 6.86% respectively which were slightly higher compared to previous studies in Ghana^8^,^19^ but lower than some other Sub-Saharan African countries^27^,^28^, and fell significantly short of the World Health Organization's global target to eliminate cervical cancer^29^. The study found significant variations in CBE and CCS uptake across different sociodemographic, lifestyle, and health related characteristics highlighting potential disparities in the access and utilization of preventive programs.

Specifically, for sociodemographic factors, the study found older women were more likely to undergo CBE and CCS compared to younger women. This finding is comparable with previous studies by Lamichhane et al^30^ who reported greater cancer screening participation among older Nepalese women and Yimer et al^28^ who reported the same among women in Sub-Saharan Africa. The higher screening among older women may be due to increased awareness and perceived risk of breast and cervical cancer among older women^8^,^15^,^31^. Older women may also have a higher utilization of healthcare services which could contribute to the increased awareness and higher cancer screening uptake^32^,^33^.

Women from wealthier households and women with higher education were found to have higher CBE and CCS uptakes in our study. This finding is documented in literature unequivocally^8^,^10^,^13^,^15^,^18^,^19^,^30^. Higher income and higher education are associated with better access to healthcare resources including preventive services and are associated with increased awareness and health literacy 14. People with higher socioeconomic backgrounds are more likely to have health insurance which increases the number of regular interactions with healthcare providers and hence better opportunities for access to screening programs^14^,^20^,^30^. In our study, health insurance was strongly associated with higher screening uptake for both breast and cervical cancer screenings. Again, health insurance reduces the financial barriers associated with healthcare access and for that matter, cancer screening services^20^.

Additionally, the study found significant rural-urban variations in CBE and CCS. Women in urban areas reported higher CBE and CCS uptake compared to women in the rural areas, similar to previous studies^13^,^15^,^30^,^34^. This difference may be explained by the disparities in healthcare access between urban and rural dwellers^15^,^34^. Urban areas typically have better healthcare infrastructure that facilitates higher screening uptake among urban dwellers in contrast to rural areas that are often faced with limited health infrastructure as well as transportation challenges and health personnel shortages thereby hindering access to preventive services^35^. Rural dwellers turn to live further from the few healthcare providers which further decreases access. In our study, the farther the women were from the nearest health facility, the less likely they were to undergo cancer screening further supporting how access to healthcare influences uptake of preventive services.

Finally, the study reported women who identified as Christians had higher CBE and CCS uptake compared to women who identified as other religions (except Islam where the difference was comparable) as previously observed^10^,^30^. This difference may reflect the cultural taboos surrounding reproductive health topics particularly for cervical cancer which has some social desirability implications^10^,^30^. This may hold true as our study also found only 1.71% of the women reported having multiple sex partners and was not a significant predictor of CBE or CCS uptake. This finding highlights the potential influence of cultural beliefs on health seeking behaviors and the need to engage religious leaders in promoting awareness and encouraging the utilization of preventive services.

This study reported significant findings that hold important implications for cancer screening and more broadly for public health interventions. The study highlighted the need for targeted efforts to address socioeconomic, geographical, and sociocultural barriers that impact access to public health programs, particularly in underserved populations. Implementing community-based outreach programs such as mobile screening exercises and health education campaigns targeted at low-income and low-educated rural dwellers may help address these barriers. Again, interventions that are tailored towards promoting health-seeking behaviors and collaborations with faith-based leaders may challenge individuals to prioritize preventive care and overcome sociocultural barriers to cancer screening.

Although the study findings provide valuable public health benefits, there are some limitations that need mentioning. First, due to the cross-sectional design of the Ghana Demographic and Health Survey, we are not able to establish temporal relationships between the studied variables. For example, we cannot establish women were residing in their current residence (rural/urban) before their cancer screening status and vice versa. The data were also self-report raising issues of recall bias and social desirability report bias. The findings should therefore be interpreted in the context of these limitations. Future research that is longitudinal in design and that objectively measures cancer screening uptake may provide robust evidence to better understand factors that influence people's cancer screening behaviors.

Despite the limitations, to the best of our knowledge, this is the first population level study in Ghana that examines predictors of cancer screening behaviors. The findings of this study are generalizable to Ghanaian women between 25 to 49 years given the robust study design.

## Conclusion

The study highlighted how cancer screening behaviors among women are influenced by the complex relationship between socio-demographic, socioeconomic, and sociocultural factors. Addressing the breast and cervical cancer screening challenges will require intentional efforts that are tailored towards improving healthcare access and increasing utilization of preventive services among underserved populations. Tailoring policies to address the poverty burden will likely lead to an increase in breast and cervical cancer screening uptakes and potentially better health outcomes.

## Data Availability

The data for this study is publicly available through the Demographic and Health Survey Program (https://dhsprogram.com/data/available-datasets.cfm) after submitting an abstract and it is approved.
